# Genetic Causal Association Between Skin Microbiota and Biological Aging: Evidence From a Mendelian Randomization Analysis

**DOI:** 10.1111/jocd.16762

**Published:** 2025-01-03

**Authors:** Yuan Li, Liwen Ma, Lipan Fan, Chuyan Wu, Dan Luo, Feng Jiang

**Affiliations:** ^1^ Department of Dermatology The Fifth People's Hospital of Hainan Province Haikou China; ^2^ Department of Dermatology Nanjing Drum Tower Hospital Clinical College of Nanjing Medical University Nanjing China; ^3^ Department of Dermatology The First Affiliated Hospital of Nanjing Medical University Nanjing China; ^4^ Department of Dermatology Chinese Academy of Sciences Zhong Guan Cun Hospital Beijing China; ^5^ Department of Rehabilitation Medicine The First Affiliated Hospital of Nanjing Medical University Nanjing China; ^6^ Department of Neonatology Obstetrics and Gynecology Hospital of Fudan University Shanghai China

**Keywords:** biological aging, causal relationship, GWAS, Mendelian randomization, skin microbiota

## Abstract

**Background:**

The skin microbiota, a complex community of microorganisms residing on the skin, plays a crucial role in maintaining skin health and overall homeostasis. Recent research has suggested that alterations in the composition and function of the skin microbiota may influence the aging process. However, the causal relationships between specific skin microbiota and biological aging remain unclear. Mendelian randomization (MR) analysis provides a powerful tool to explore these causal links by utilizing genetic variants as instrumental variables, thereby minimizing confounding factors and reverse causality that often complicate observational studies.

**Methods:**

We utilized a two‐sample MR approach with population‐based cross‐sectional data from two German cohorts, KORA FF4 (*n* = 324) and PopGen (*n* = 273). In total, GWAS summary data from 1656 skin samples and datasets on accelerated biological age were analyzed to investigate the causal relationship between skin microbiota and accelerated biological aging. The primary analysis was performed using the inverse variance weighted (IVW) method with random effects and was further supported by MR‐Egger regression, Cochran's *Q* test, and a range of sensitivity analyses.

**Results:**

The MR analysis revealed that for biological age acceleration (BioageAccel), the IVW analysis identified protective effects from certain skin microbiota, including Alphaproteobacteria_Dry (*p* = 0.046), Asv033_sebaceous (*p* = 0.043), Burkholderiales_Moist (*p* = 0.008), and Proteobacteria_Moist (*p* = 0.042). Similar protective effects were observed for Burkholderiales_Moist (*p* = 0.045) and Proteobacteria_Moist (*p* = 0.012) in the weighted median analysis. In contrast, Paracoccus_Moist (*p* = 0.013) and Proteobacteria_Sebaceous (*p* = 0.005) were associated with accelerated aging. When using PhenoAge acceleration as the outcome, the IVW analysis linked skin microbiota like Asv005_Dry (*p* = 0.026), ASV039_Dry (*p* = 0.003), Betaproteobacteria_Sebaceous (*p* = 0.038), and Chryseobacterium_Moist (*p* = 0.013) with accelerated aging. The weighted median analysis supported these findings and also identified protective effects from ASV011_Dry (*p* = 0.021), ASV023_Dry (*p* = 0.040), Bacteroidales_Dry (*p* = 0.022), Enhydrobacter_Moist (*p* = 0.038), Proteobacteria_Moist (*p* = 0.002), and Rothia_Moist (*p* = 0.038).

**Conclusions:**

This two‐sample MR study reveals potential causal relationships between skin microbiota and aging. However, to confirm these findings, further randomized controlled trials (RCTs) are necessary.

## 1 Introduction

Recent studies have identified the skin as the second most microbiome‐rich organ in the human body, following the gastrointestinal tract, with considerable inter‐individual variability [1]. The skin microbiota, which includes bacteria, fungi, viruses, and other microorganisms, forms a complex ecosystem with the host's skin cells and plays a vital role in maintaining skin health and function. Emerging research has underscored the significant role of skin microbiota in biological aging [2]. As individuals age, the diversity and abundance of their skin microbiota tend to decline, which can negatively affect skin barrier function and immune response [3]. In older adults, this reduction in microbial diversity is often accompanied by the proliferation of harmful microorganisms, such as *Propionibacterium* and certain fungi, which may be linked to skin lesions and chronic inflammation. Aging is also associated with impaired skin barrier function and immune system degradation, which can disrupt skin microbiome–host interactions, leading to an increased risk of skin health issues. Moreover, the aging process is frequently marked by a state of chronic low‐grade inflammation, known as “inflammaging,” which may be driven by dysbiosis of the skin microbiota, thereby accelerating the aging of the skin [4, 5].

Biological age is determined by evaluating a range of biomarkers, health indicators, and biological profiles, rather than being solely based on chronological age, which is defined by the passage of time. Unlike chronological age, biological age offers deeper insights into the aging process, providing a more comprehensive and dynamic understanding of aging [6]. In this study, we used both biological and chronological age metrics to assess aging. The first metric, PhenoAge, is derived from chronological age and incorporates factors such as white blood cell count, alkaline phosphatase, glucose levels, total body fluid volume, and leukocyte count. The second metric, Biological Age (BioAge), also relies on chronological age and shares some components with PhenoAge, including albumin, creatinine, and alkaline phosphatase. In addition, BioAge includes glycosylated hemoglobin, serum blood pressure, and total cholesterol. Previous studies have shown that both of these aging indices are reliable predictors of aging‐related outcomes [7, 8].

The skin plays a vital role in protecting internal organs, regulating body temperature, and hosting a rich microbiota, collectively known as the skin microbiota [9]. Together with skin cells, these microorganisms form a complex and dynamic ecosystem that significantly influences skin health and function [10]. Recent advancements in biological and medical research have highlighted the critical role of skin microbiota in the process of biological aging. As individuals age, the composition and function of the skin microbiota undergo substantial changes, impacting not only the skin's appearance and structure but also its association with age‐related diseases and inflammation [11, 12]. Exploring the relationship between skin microbiota and biological aging sheds light on the underlying mechanisms of aging and provides scientific evidence and innovative approaches for developing new anti‐aging strategies. Mendelian randomization (MR) serves as a powerful tool in this area of research [13].

In this study, we employ Mendelian randomization to explore the causal relationship between skin microbiota and biological aging, investigating whether specific skin microbiota profiles might accelerate or decelerate the aging process. The goal of this research is to provide new insights into promoting healthy aging by modulating the skin microbiota.

## 2 Materials and Methods

### 2.1 Study Design

**FIGURE 1:**
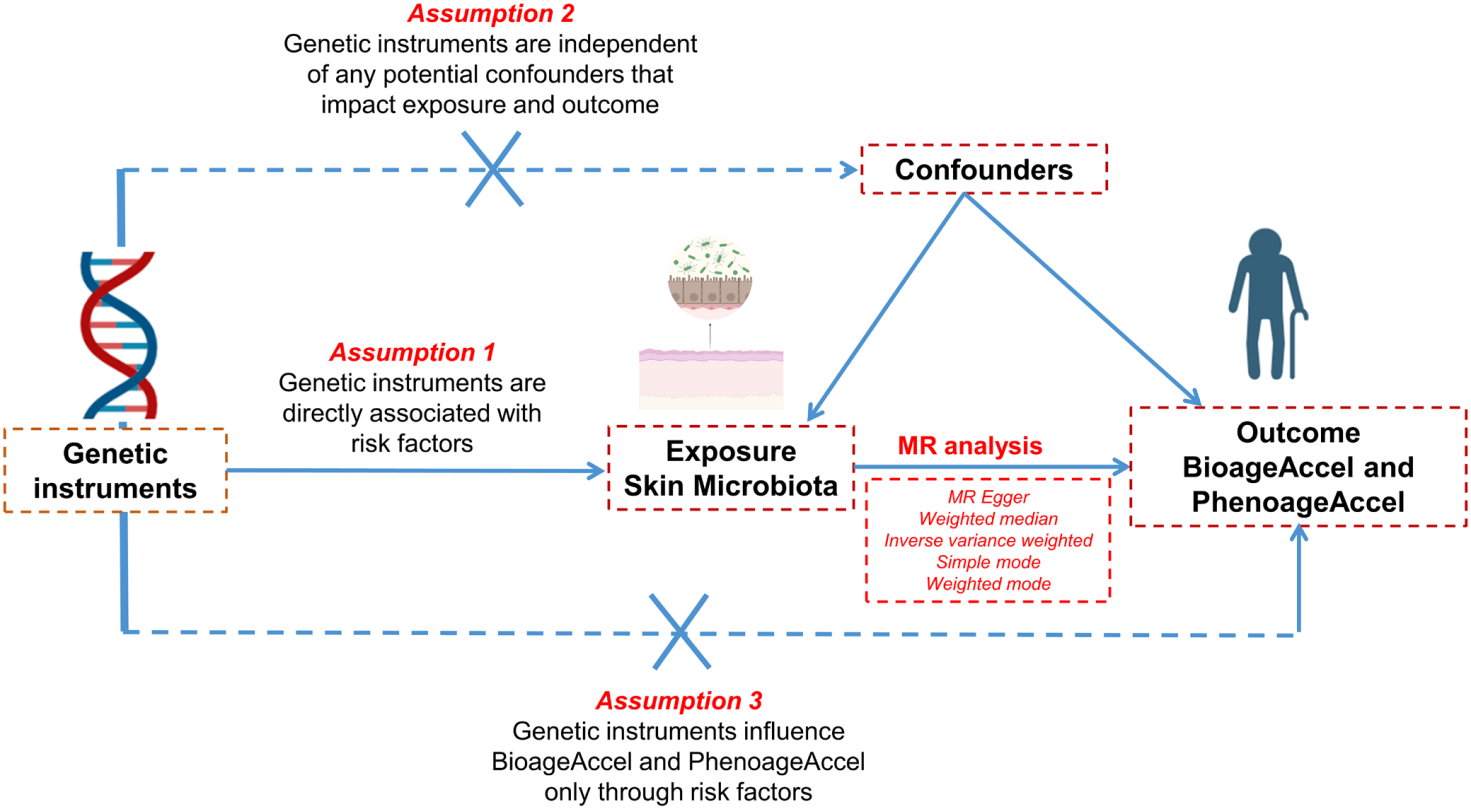
Overview of the design and methods used in this MR study.

Our study design is depicted in Figure 1. We utilized a two‐sample MR approach to examine the relationship between skin microbiota and biological aging. This study builds on the skin microbiota research conducted by Lucas et al. and the biological aging study by Kuo et al. [6, 14]. Importantly, there was no overlap between the exposure and outcome samples within the context of the genome‐wide association studies (GWAS) data. Each study received approval from the respective ethics committees.

### 2.2 Data Sources

The GWAS summary data for the skin microbiota were derived from two population‐based cross‐sectional German cohorts, KORA FF4 (*n* = 324) and PopGen (*n* = 273), comprising a total of 1656 skin samples. These samples were collected from different skin microenvironments: Dry areas (dorsal and palmar surfaces of the forearm from PopGen), moist areas (antecubital fossae from both KORA FF4 and PopGen), and sebaceous‐rich areas (postauricular folds from KORA FF4 and forehead from PopGen). The microbiota profiles were determined by sequencing the V1–V2 regions of the 16S ribosomal RNA (rRNA) gene.

The GWAS summary data for biological age metrics were sourced from Kuo et al.'s [6] study, which features PhenoAge‐acceleration (PhenoAgeAccel) and BioAge‐acceleration (BioAgeAccel) data derived from 107 460 and 98 446 individuals of European descent, respectively. Both metrics, PhenoAge and BioAge, have been validated as reliable indicators for predicting biological age. Levine et al. developed these predictors using data from the National Health and Nutrition Examination Survey (NHANES) III [7, 8]. For detailed information on the formulas and biomarkers used to calculate PhenoAge and BioAge, refer to the paper by Kuo et al., who also validated these measures against data from the UK Biobank. The two indicators of biological age were calculated using linear regression modeling, where the residuals from PhenoAge and BioAge were used to estimate chronological age and then to predict the biological age of the individuals. Details of studies and datasets used for analyses can be found in Table S1.

### 2.3 Instrumental Variables

We analyzed the summary data using a two‐sample MR approach, employing genetic variants as instrumental variables (IVs) in line with MR principles. These IVs had to satisfy three key criteria: (1) they must be associated with the skin microbiota; (2) they must be independent of any confounding factors; and (3) they must influence the aging process solely through their effect on the skin microbiota, without involvement in other indirect pathways. In this study, we initially considered the skin microbiota as the exposure variable to assess its causal impact on aging. To enhance the robustness of our sensitivity analyses, we referenced prior MR studies on skin microbiota and applied a stringent threshold of *p* < 1 × 10^−5^ for selecting single‐nucleotide polymorphisms (SNPs) [15, 16]. The IVs used in the MR analysis of the association between skin microbiota and biological aging (BioAge and PhenoAge) are detailed in Tables S2 and S3.

### 2.4 MR Analysis

MR analysis uses genetic variants as IVs to estimate the causal effect of an exposure on an outcome, thereby minimizing confounding factors and reverse causality that are typically present in observational studies. We selected SNPs associated with skin microbiota as instrumental variables based on GWAS. We employed several MR methods to assess the causal effects, each with different strengths in handling pleiotropy or heterogeneity [17, 18]. The inverse variance‐weighted (IVW) method was used as the primary analysis approach, assuming that all SNPs are valid instruments. This method meta‐analyzes the ratio estimates of individual SNPs to provide an overall causal estimate. We also applied the MR‐Egger regression method to assess potential pleiotropy, with the MR‐Egger intercept indicating the presence of directional pleiotropy. Unlike IVW, MR‐Egger allows for the possibility that some genetic variants may influence the outcome through pathways other than the exposure. The weighted median method was also used, which provides a consistent estimate of the causal effect even if some of the instrumental variables are invalid [19, 20, 21].

The Cochran's *Q* statistic was calculated to assess heterogeneity, representing the sum of the weighted squared differences between individual SNP estimates and the overall IVW estimate. A large *Q* statistic indicates greater variability in the effect estimates than would be expected by chance alone, suggesting potential heterogeneity. We tested for pleiotropy using the MR‐Egger intercept, where a *p*‐value of < 0.05 was considered indicative of potential causal significance. All statistical analyses were conducted using R (version 4.2.2). To assess the sensitivity of our results, we employed the leave‐one‐out method. Similar to a meta‐analysis, this approach involves sequentially removing each SNP and recalculating the effect estimates using the IVW method for the remaining SNPs. This process helps to identify the influence of individual SNPs on the overall causal inference.

## 3 Results

**FIGURE 2:**
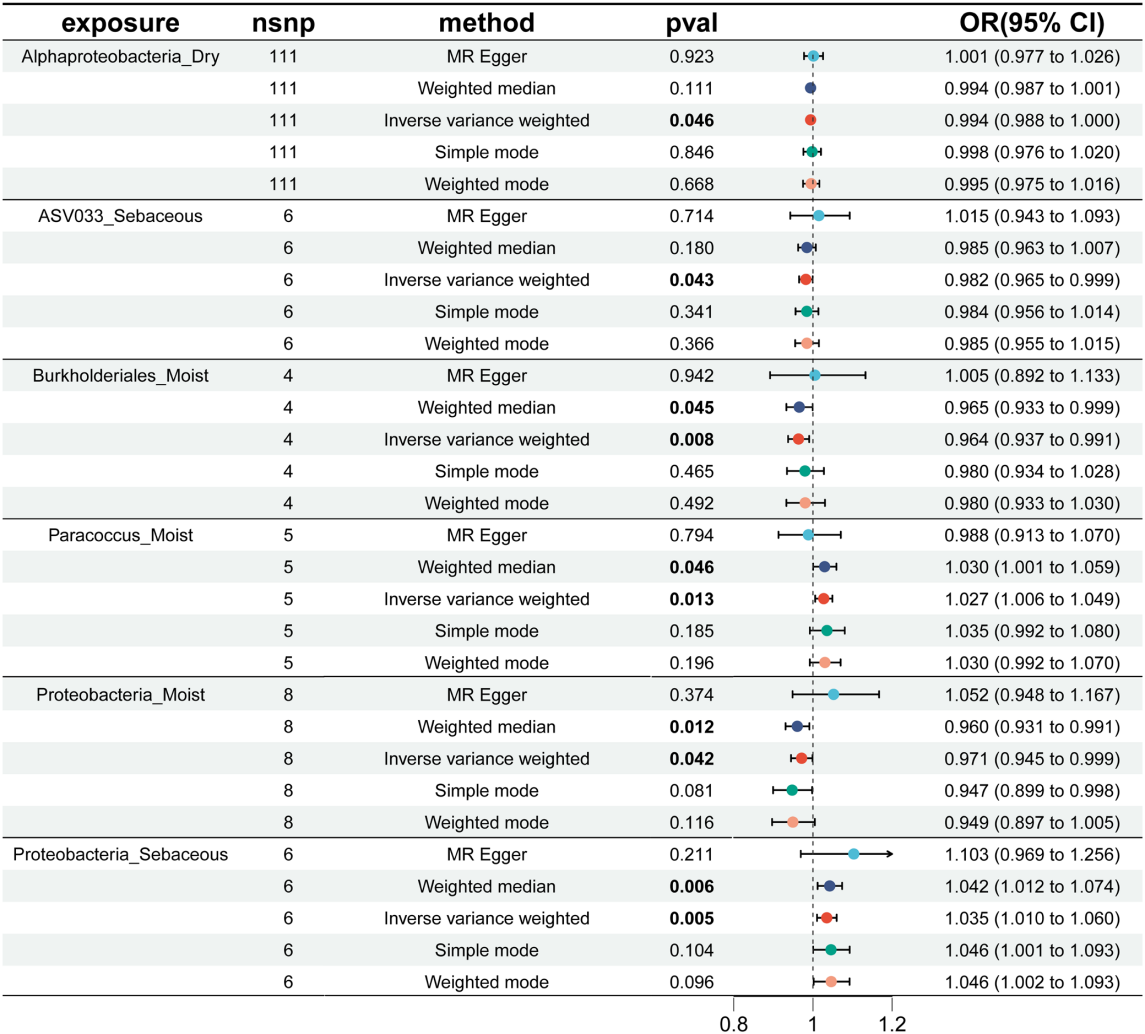
MR results of causal relationships between skin microbiota and Bioage acceleration. Bolded numbers represent *p* < 0.05.

For BioAgeAccel, the IVW analysis revealed that certain skin microbiota exhibited protective effects: Alphaproteobacteria_Dry (*p* = 0.046), Asv033_sebaceous (*p* = 0.043), Burkholderiales_Moist (*p* = 0.008), and Proteobacteria_Moist (*p* = 0.042). Similarly, in the weighted median analysis, Burkholderiales_Moist (*p* = 0.045) and Proteobacteria_Moist (*p* = 0.012) also showed protective effects (see Figure 2, Table S4). On the other hand, the IVW analysis indicated that Paracoccus_Moist (*p* = 0.013) and Proteobacteria_Sebaceous (*p* = 0.005) were associated with accelerated aging; these findings were supported by the weighted median analysis, which also found that Paracoccus_Moist (*p* = 0.046) and Proteobacteria_Sebaceous (*p* = 0.006) accelerated aging (see Figure 2, Table S4).

**FIGURE 3:**
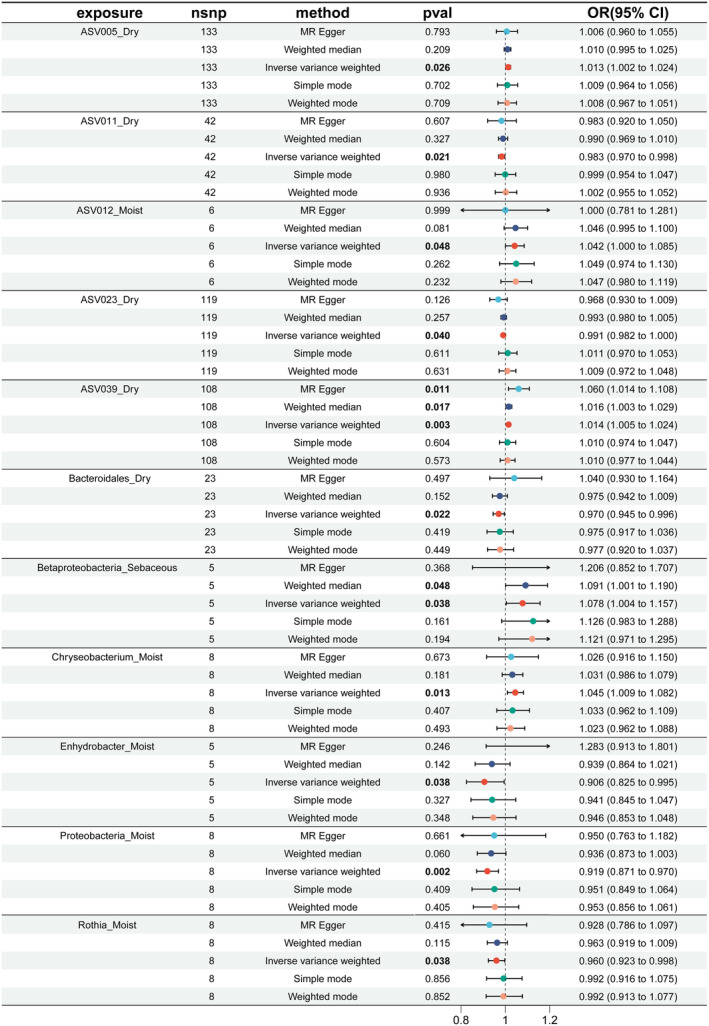
MR results of causal relationships between skin microbiota and PhenoAge acceleration. Bolded numbers represent *p* < 0.05.

With PhenoAge as the outcome parameter, the IVW analysis identified several skin microbiota associated with accelerated aging: Asv005_Dry (*p* = 0.026), ASV039_Dry (*p* = 0.003), Betaproteobacteria_Sebaceous (*p* = 0.038), Chryseobacterium_Moist (*p* = 0.013), and ASV012_Moist (*p* = 0.048) (see Figure 3, Table S5). In the weighted median analysis, ASV039_Dry (*p* = 0.017) and Betaproteobacteria_Sebaceous (*p* = 0.048) also suggested associations with accelerated aging. Conversely, ASV011_Dry (*p* = 0.021), ASV023_Dry (*p* = 0.040), Bacteroidales_Dry (*p* = 0.022), Enhydrobacter_Moist (*p* = 0.038), Proteobacteria_Moist (*p* = 0.002), and Rothia_Moist (*p* = 0.038) exhibited protective effects (see Figure 3, Table S5).

**FIGURE 4:**
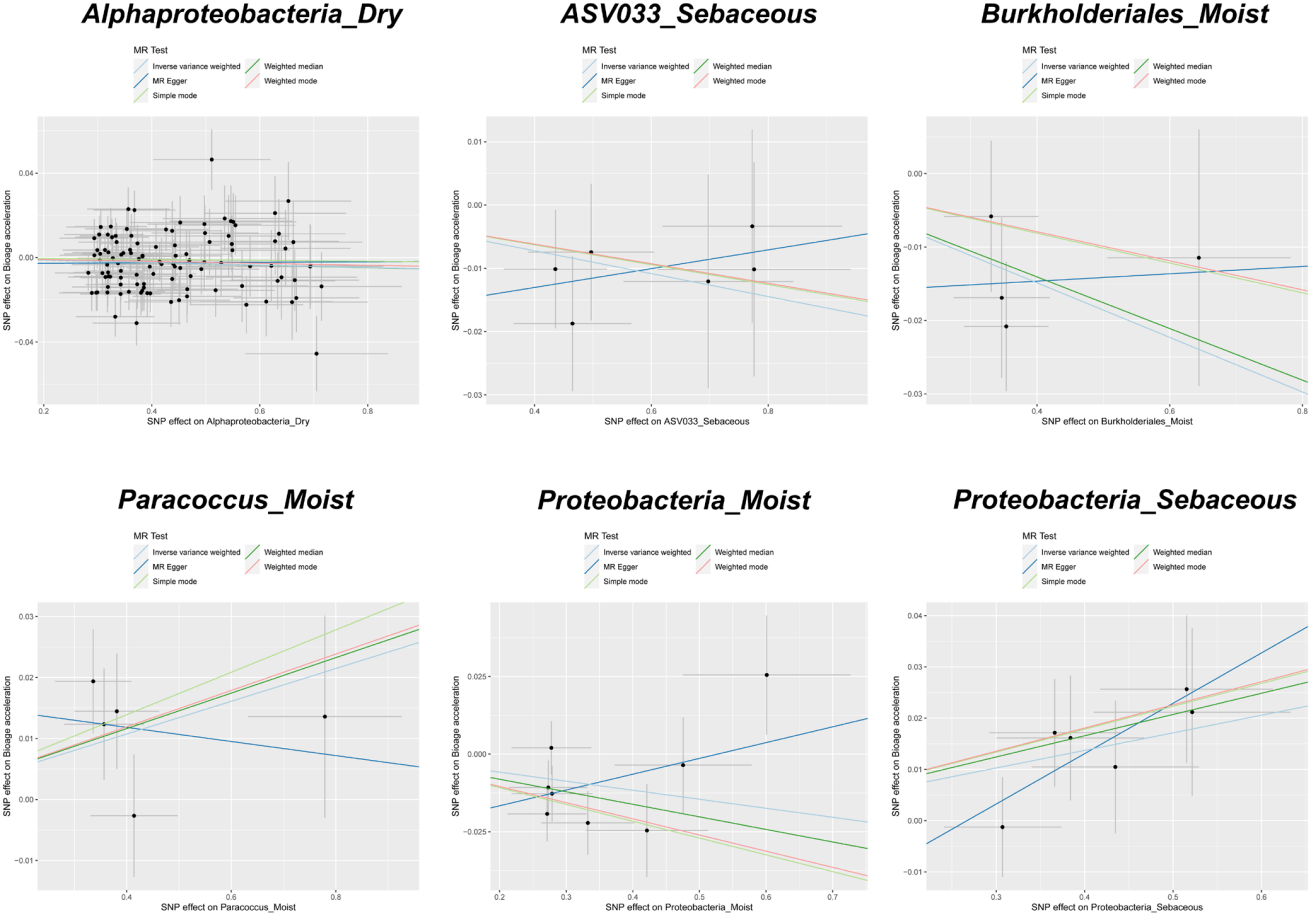
Scatter plots for the causal relationships between skin microbiota and Bioage acceleration.

**FIGURE 5:**
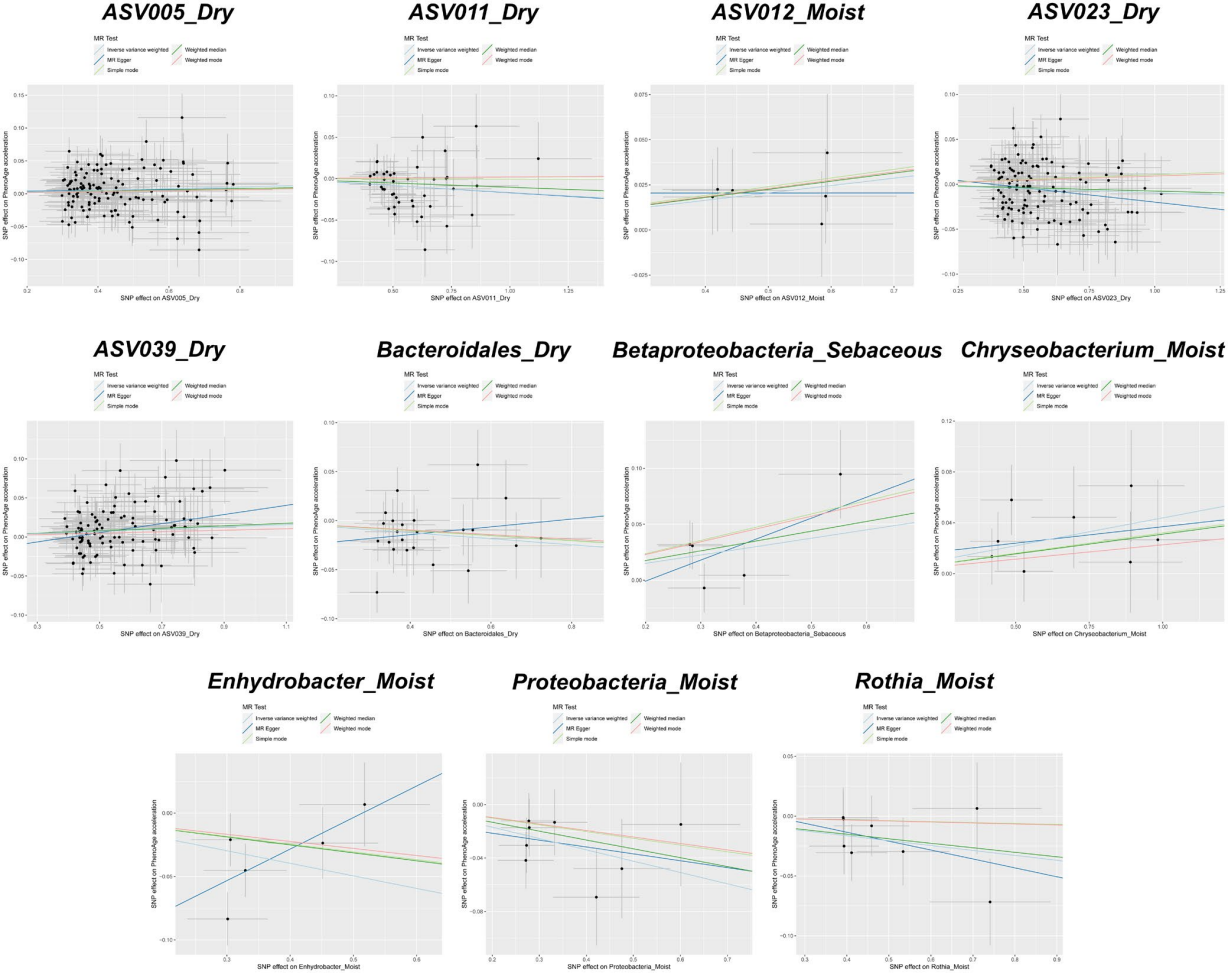
Scatter plots for the causal relationships between skin microbiota and PhenoAge acceleration.

**FIGURE 6:**
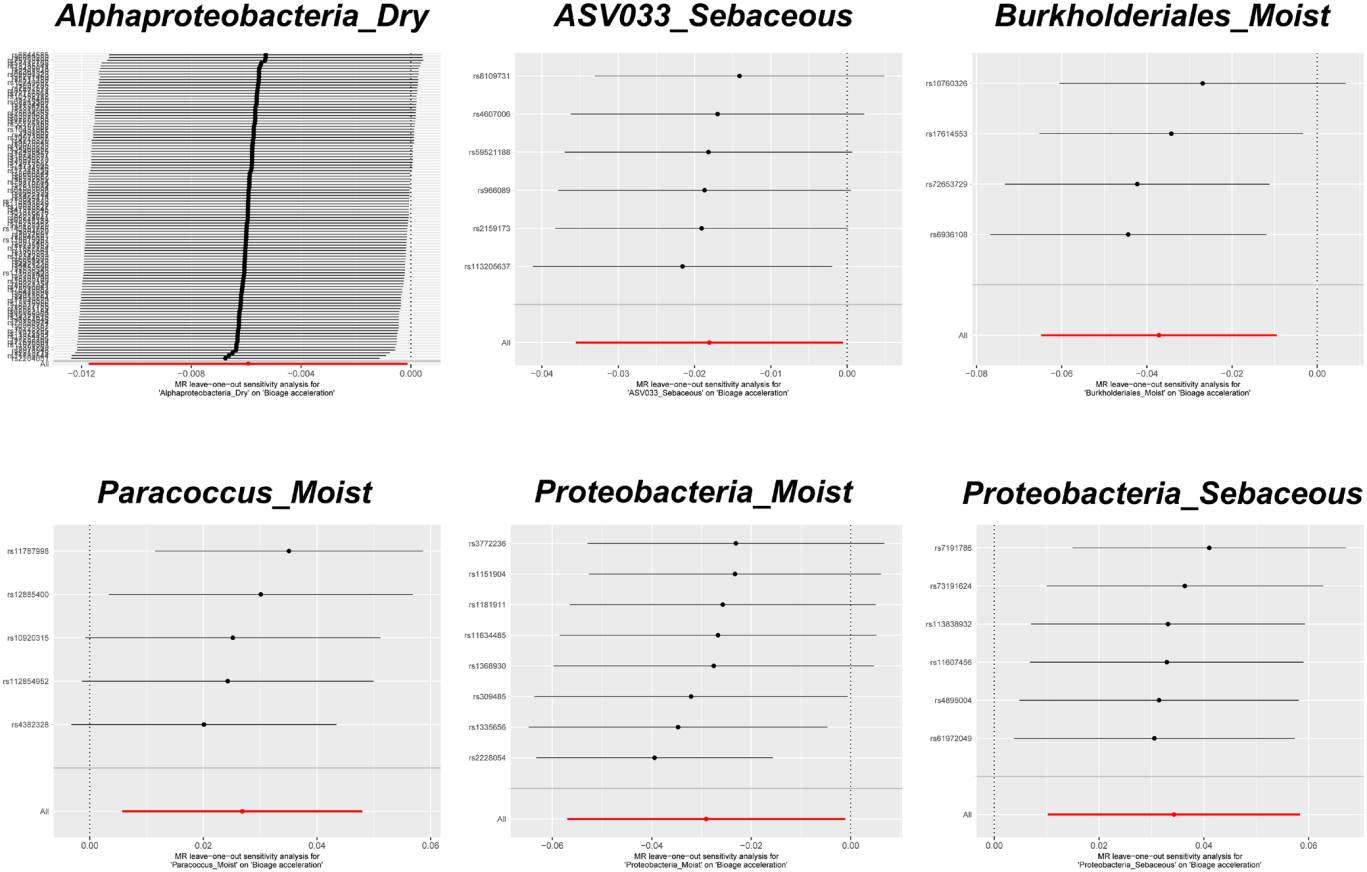
Scatter plots for the causal relationships between skin microbiota and Bioage acceleration.

**FIGURE 7:**
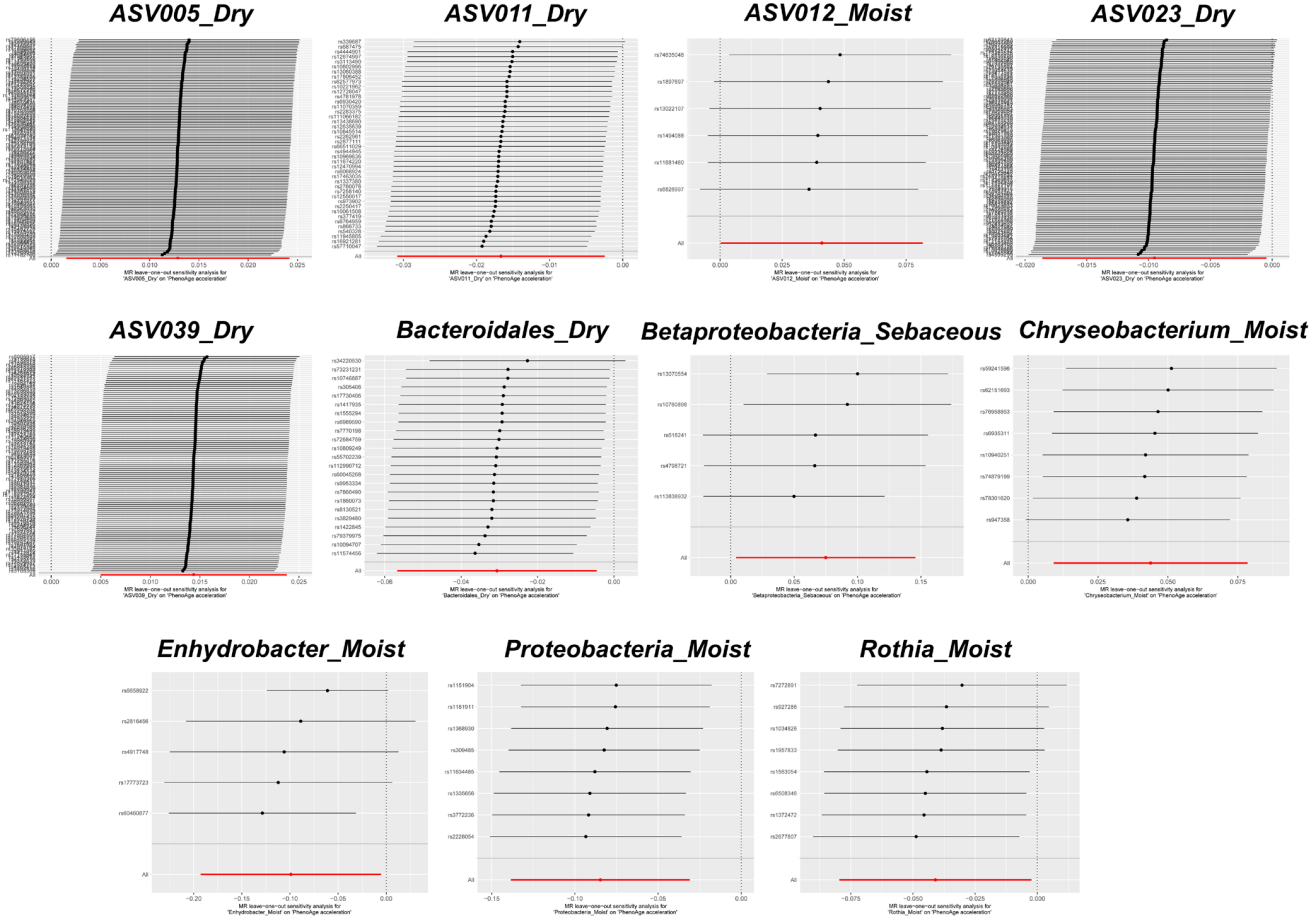
Scatter plots for the causal relationships between skin microbiota and PhenoAge acceleration.

All the aforementioned skin microbiota groups demonstrated evidence of causality or indicative causality. However, when BioAgeAccel was used as the outcome variable, significant heterogeneity was observed only in Alphaproteobacteria_Dry (‘Cochran's IVW *Q*’ = 146.95, *p* = 0.010; ‘Cochran's MR‐Egger *Q*’ = 146.47, *p* = 0.009), whereas the other microbiota groups did not show substantial heterogeneity in either Cochran's IVW *Q* or Cochran's MR‐Egger *Q* tests (see Figure 4, Table S6). Similarly, when PhenoAgeAccel was the outcome variable, only Enhydrobacter_Moist exhibited significant heterogeneity (‘Cochran's IVW *Q*’ = 10.76, *p* = 0.029; ‘Cochran's MR‐Egger *Q*’ = 4.47, *p* = 0.21), while the Cochran's IVW *Q* and MR‐Egger *Q* values for the other microbiota groups did not suggest considerable heterogeneity (see Figure 5, Table S7). The MR‐Egger regression intercept analysis did not reveal any significant directional pleiotropy (Tables S8 and S9). Further leave‐one‐out analyses did not identify any significantly outlying SNPs (see Figures 6 and 7), reinforcing the reliability of the MR estimates and indicating that our findings are not biased by directional pleiotropy. The leave‐one‐out results also confirmed that no single SNP had a decisive influence on the causal inference.

## 4 Discussion

The results of this study suggest that several skin microbiota may be associated with delayed aging, including ASV011_Dry, ASV023_Dry, Bacteroidales_Dry, Enhydrobacter_Moist, Proteobacteria_Moist, Rothia_Moist, Alphaproteobacteria_Dry, Asv033_sebaceous, and Burkholderiales_Moist. Specifically, Alphaproteobacteria_Dry has been found to exhibit antioxidant enzyme activity, which can neutralize free radicals and reduce oxidative stress on the skin, thereby helping to mitigate skin aging caused by UV exposure and environmental pollution [22]. Asv033_sebaceous regulates sebum secretion and metabolism, maintaining the skin's oil–water balance, preventing excessive dryness or oiliness, and preserving skin elasticity and smoothness. Burkholderiales_Moist shows anti‐inflammatory properties, which can reduce chronic inflammatory responses and are crucial for skin protection [23]. Proteobacteria_Moist may help maintain the skin barrier function in moist skin areas by producing metabolic products or enzymes that prevent the intrusion of harmful external substances [24]. ASV011_Dry and ASV023_Dry help retain skin moisture, preventing dryness and thereby slowing the formation of fine lines and wrinkles [25]. Bacteroidales_Dry may modulate the skin's immune system and reduce inflammatory responses, thus preventing age‐related skin inflammation [26]. Enhydrobacter_Moist can produce antimicrobial peptides or other substances that inhibit pathogens, preventing skin infections and related inflammatory responses [27]. Rothia_Moist balances the skin microbiome by inhibiting the excessive growth of harmful bacteria and maintaining skin health [28]. These microbiota may help combat biological aging of the skin through various mechanisms, such as antioxidant protection, anti‐inflammatory effects, maintaining skin moisture and barrier function, regulating sebum metabolism, and balancing the microbiome. Understanding these mechanisms will aid in developing skincare strategies for skin health and anti‐aging.

The following microbiota are suggestively associated with accelerated aging: Asv005_Dry, ASV039_Dry, Betaproteobacteria_Sebaceous, Chryseobacterium_Moist, ASV012_Moist, Paracoccus_Moist, and Proteobacteria_Sebaceous. These microbiota may contribute to skin aging through various mechanisms. Asv005_Dry and ASV039_Dry, present in dry skin areas, may lead to decreased skin barrier function and moisture loss, thereby accelerating aging by disrupting the skin barrier or inducing localized inflammation [29]. Betaproteobacteria_Sebaceous is linked to the dysregulation of sebum metabolism, which can result in sebum oxidation and inflammatory responses. The oxidation products from excessive sebum oxidation are critical factors in skin aging, causing cellular damage, DNA mutations, and collagen degradation, leading to wrinkles and reduced skin elasticity [30]. Chryseobacterium_Moist may proliferate excessively in moist environments, producing inflammatory metabolites or toxins that accelerate skin aging and disrupt the balance of beneficial microorganisms, thereby weakening the skin's natural defense mechanisms [31]. ASV012_Moist might impair the skin barrier and increase inflammation in moist environments, accelerating aging through dysbiosis and the growth of harmful bacteria [32]. Paracoccus_Moist may promote skin aging by producing oxidative products and inducing inflammatory responses, leading to increased oxidative stress and immune dysregulation [33]. Proteobacteria_Sebaceous, in sebaceous regions, may affect skin cell function and metabolism through lipid metabolism and oxidation reactions, with metabolites such as lipopolysaccharides and short‐chain fatty acids potentially accelerating skin aging. Lipopolysaccharides, as potent inflammatory agents, may exacerbate chronic inflammation in the skin [34]. These microbiota may accelerate skin aging through mechanisms such as oxidative stress, inflammation, disruption of the skin barrier, and microbial imbalance. Further research and clinical case studies are essential to fully understand how these microbiota impact skin health and aging.

In this research, we used BioAgeAccel and PhenoAgeAccel as metrics to characterize findings related to accelerated aging [35]. Previous studies have demonstrated that BioAge and PhenoAge are effective predictors of aging outcomes, with various studies employing these metrics to describe biological age and aging acceleration [36, 37, 38, 39]. For example, genome‐wide association studies by Kuo et al. found that BioAgeAccel and PhenoAgeAccel were linked to coronary and meteorological risks, as well as immune response inhibition, and were strongly associated with aging. Therefore, we consider BioAgeAccel and PhenoAgeAccel to be practical tools for quantifying the impact of the skin microbiome on accelerated aging.

Existing research indicates a close relationship between skin microbiota dysbiosis and aging. Exploratory studies by Edda Russo et al. [2] have revealed significant differences in the structure of facial skin microbiota among women of different ages. Furthermore, the functional and associative interactions between microbiota and host genetic factors may influence pathways related to aging, such as reactive oxygen species (ROS) damage repair and collagen metabolism. A study by Shi Huang tested the ability of oral, gut, and skin (hand and forehead) microbiota to predict adult age. Using random forest regression and integrating data from multiple public studies, the model results from individual cohorts demonstrated that the skin microbiota provided the most accurate age prediction. These findings suggest that microbiota may interact with ultraviolet radiation, potentially influencing the occurrence or prevention of skin damage and skin cancer. This knowledge opens new avenues for modulating the microbiome to sustain or enhance wellness throughout the aging process. Consequently, probiotics, both topical and oral, may emerge as promising treatments for preventing premature skin aging.

This research offers several notable benefits. First, it employed a two‐sample MR approach, which helped mitigate bias from pooled data on exposures and outcomes, as well as from confounding factors. Second, the study utilized multiple statistical methods for MR analyses, thereby minimizing the impact of horizontal pleiotropy. Additionally, the research leveraged summary data from the most significant published multi‐cohort GWAS on skin microbiota, reducing biases due to methodological variations in sampling the skin biome and ensuring the representativeness of the outcomes and the validity of the IVs used in the MR analyses. Finally, the exposure and outcome samples used in the research did not overlap, further decreasing the likelihood that weak instrument bias would lead to Type I errors [40].

Although this investigation provides valuable insights, it also has some limitations. First, the research predominantly focuses on cutaneous microbiomes that are strongly correlated with genomic mutations, potentially overlooking skin microbiomes with weaker relationships to individual genetic variation. This gap suggests the need for further exploration using alternative investigative approaches. Second, due to the constraints of the existing aggregated data from skin microbiome GWAS, this study only included microbiome data at the genus level, limiting the analysis of aging effects on the skin microbiome at the species‐specific level. To incorporate more instrumental variations into susceptibility analyses and cross‐sectional polymorphism tests, the study adopted a lower SNP threshold than the conventional GWAS significance level (*p* < 5 × 10^−8^), which may introduce additional complexities. Moreover, senescence is a complex process influenced by multiple factors. While BioAge and PhenoAge capture certain aspects of aging, a comprehensive understanding requires validation through GWAS studies with larger sample sizes and the inclusion of more observed metrics. The predictive capability of biological age as an indicator of aging may vary depending on the population, methodology, and datasets used, making it difficult to identify universally applicable aging markers or processes. Variations in genetic diversity, physical health, and exposure to environmental factors further complicate the identification of prevalent aging‐related markers. Lastly, since the GWAS data related to the skin microbiota are predominantly derived from European populations, further investigation and verification are necessary to examine the causes and effects of the relationship between skin microbiota and accelerated aging in non‐European populations.

The causal link between skin microbiomes and aging remains a significant area of inquiry. However, large‐scale randomized controlled trials (RCTs) have encountered numerous challenges in this field, necessitating alternative research methods to clarify the causal connections between specific microbiomes and the aging process. MR analyses offer a promising approach for uncovering these causal relationships. To deepen our understanding, future research should focus on elucidating how skin microbiota influence aging through specific molecular pathways and interactions. Understanding these mechanisms will clarify the precise role of skin microbiota in biological aging and may provide a foundation for developing strategies to promote healthy aging and prevent age‐related diseases. Integrating MR analysis results with additional comprehensive datasets, such as morphogenomics, metallomics, and transcriptomics, could provide a more complete picture of the complex interactions between the skin microbiome and biological aging. This knowledge could lead to the development of personalized therapeutic measures aimed at optimizing the composition of the skin's microbiota to promote healthier aging trajectories. Such research holds the potential to identify effective strategies for delaying aging, improving skin health, and enhancing the quality of life for older individuals.

## 5 Conclusions

This two‐sample Mendelian randomization study reveals a potential causal relationship between skin microbiota and aging. However, further RCTs are needed to validate these findings. Future RCTs could help establish specific causal pathways, providing scientific evidence and new strategies for researching anti‐aging treatments. Such studies are expected to lead to breakthroughs in skin health and aging management and to optimize personalized interventions to promote a healthy aging process.

## Author Contributions

Yuan Li and Liwen Ma conceived and designed the study, conducted the data analysis, and drafted the manuscript. Chuyan Wu assisted with data analysis and contributed to manuscript preparation. Lipan Fan provided essential resources. Feng Jiang and Dan Luo provided guidance on the study's methodology, assisted with the interpretation of the results, and contributed to manuscript preparation and revision. All authors read and approved the final manuscript.

## Ethics Statement

These datasets have already obtained appropriate ethical approvals and informed consent in their original studies. Therefore, this research does not involve the collection of new human data and does not require additional ethical approval. These datasets have been widely used in scientific research and comply with ethical and legal requirements.

## Consent

The authors have nothing to report.

## Conflicts of Interest

The authors declare no conflicts of interest.

## Supporting information


**Table S1** Overview of studies and datasets utilized in the analyses.

**Table S2** Genetic instruments used in the MR analysis for the relationship between skin microbiota and BioAgeAccel.

**Table S3** Genetic instruments used in the MR analysis for the relationship between skin microbiota and PhenoAgeAccel.

**Table S4** Comprehensive results of MR estimates concerning the association between skin microbiota and BioAgeAccel.

**Table S5** Comprehensive results of MR estimates concerning the association between skin microbiota and PhenoAgeAccel.

**Table S6** Assessment of heterogeneity among instrumental variables for skin microbiota (BioAgeAccel).

**Table S7** Assessment of heterogeneity among instrumental variables for skin microbiota (PhenoAgeAccel).

**Table S8** Evaluation of directional horizontal pleiotropy using the intercept term in MR‐Egger regression for the association between skin microbiota and BioAgeAccel.

**Table S9** Evaluation of directional horizontal pleiotropy using the intercept term in MR‐Egger regression for the association between skin microbiota and PhenoAgeAccel.

## Data Availability

The GWAS datasets related to Skin microbiota were obtained from https://www.helmholtz‐munich.de/en/kora/for‐scientists/cooperation‐withkora/index.html, the PhenoAgeAccel datasets were obtained from https://figshare.com/ndownloader/files/23739971, the BioAgeAccel datasets were obtained from https://figshare.com/ndownloader/files/23739935.
